# Application of the transgender male voice questionnaire in a Brazilian population sample

**DOI:** 10.3389/fpubh.2024.1480423

**Published:** 2024-12-16

**Authors:** Karine Schwarz, Anna Paula Villas-Bôas, Carla Aparecida Cielo, Dhiordan Carodoso da Silva, Eliane Dias da Silva, Tayane Muniz Fighera, Angelo Brandelli Costa, Maria Inês Rodrigues Lobato, Poli Mara Spritzer

**Affiliations:** ^1^Division of Endocrinology, Hospital de Clínicas de Porto Alegre, Porto Alegre, Rio Grande do Sul, Brazil; ^2^Department of Psychiatry, Gender Identity Program at Hospital de Clínicas de Porto Alegre, Porto Alegre, Rio Grande do Sul, Brazil; ^3^Postgraduate Program in Endocrinology and Metabology, School of Medicine, Federal University of Rio Grande do Sul, Porto Alegre, Rio Grande do Sul, Brazil; ^4^Department of Speech Therapy, Federal University of Santa Maria, Santa Maria, Rio Grande do Sul, Brazil; ^5^Department of Psychology, Pontifical Catholic University of Porto Alegre, Porto Alegre, Brazil

**Keywords:** voice, voice satisfaction, gender identity, transgender, hormone therapy

## Abstract

**Objectives:**

The aim of this study was to adapt and apply the Portuguese version of the Transgender Man Voice Questionnaire in a sample of Brazilian transgender men and to investigate the relationship between voice satisfaction and hormone therapy duration. In addition, we suggest reducing and reformulating the questionnaire for screening.

**Methods:**

We conducted a cross-sectional study of 31 transgender men aged 18–50 years undergoing hormone therapy who answered a questionnaire adapted from the Transgender Woman Voice Questionnaire, validated in Portuguese. Sociodemographic and clinical data were collected from the individuals’ electronic medical records: age, smoking status, and type and duration of hormone therapy. The questionnaire, consisting of 30 questions rated on a Likert scale, was answered individually during a psychotherapy session. In each question, the gender-specific words were modified. Furthermore, we added a question: 31 (After GAHT, my voice became completely male), with the response options yes or no. In questions 32 and 33, asking participants to provide an overall rating of their voice. Total score ranged from 0 to 120, with higher scores indicating greater dissatisfaction with voice.

**Results:**

Mean patient age was 30.13 ± 7.6 years, and 19.4% were smokers. The mean duration of hormone therapy was 29.7 ± 24.9 months, and 95% received intramuscular testosterone cypionate, maintaining serum testosterone levels within the male reference range. The questionnaire mean total score was 51 ± 17.72. There was a significant negative correlation between the questionnaire total score and duration of hormone therapy (*r* = −0.484, *p* = 0.006). The questionnaire had a high level of internal consistency/reliability, with a Cronbach’s alpha coefficient of 0.95 for all items and a split-half Spearman-Brown coefficient of 0.96. For the elaboration of a screening tool, it is suggested to remove questions 8, 10, 12, 13, 14, 17, 19, 23, 27, and 29 and modify question 1.

**Conclusion:**

Longer hormone therapy favors voice deepening and satisfaction with voice. The psychometric properties of the Transgender Man Voice Questionnaire are reliable, supporting its use as a screening tool in clinical practice and as an adjunct to the planning of vocal and communication support for transgender individuals.

## Introduction

Understanding voice satisfaction is essential for creating effective strategies and programs to support the vocal and communication needs of transgender individuals. Trans men generally benefit from hormone therapy (HT) and many do not seek care for voice improvement (1–4).

Although HT is effective for lowering the fundamental frequency of the voice (f0) in transgender men, it is estimated that not all individuals are completely satisfied with their voices (1, 2, 4–8). There are reports of a decrease f0 after 12 months of HT and evidence that this population may face problems related to vocal timbre, decrease in frequency, vocal power, vocal control and stability, glottic function, amplitude of pitch and vocal quality (1, 4, 6, 8). Authors report that problems in f0 lowering can be expected in about 10% of individuals and seem, at least in part, to be associated with decreased androgen sensitivity (3). Ziegler et al. (9) found in their meta-analysis of 19 studies that among individuals undergoing at least 1 year of testosterone therapy, 21% did not achieve cisgender male normative vocal frequencies, 21% reported incomplete congruence between voice and gender or experienced voice-related issues, and 16% expressed dissatisfaction with their voice.

It is known that increasing vocal gender congruence improves quality of life and reduces anxiety and depression levels in transgender men (10). Thus, the importance of a first speech-language pathology assessment, as well as the application of a voice satisfaction questionnaire in transgender men, should be considered (2, 5).

Furthermore, when using a vocal satisfaction questionnaire, it is important to consider that some transgender and gender-diverse individuals may not have access to, or may choose not to undergo, testosterone treatment but still wish to alter their voice. Additionally, there are those who may be dissatisfied with the results of testosterone treatment (2).

The Transsexual Voice Questionnaire for Male-to-Female Transsexuals (TVQ^MtF^), now renamed as the Trans Woman Voice Questionnaire, was validated with a tool designed to measure the perceptions of transgender women regarding their voice (11). Voice pitch was identified as the variable with the greatest level of dissatisfaction, and voice complaints were directly related to psychosocial issues. The voice satisfaction questionnaire for transgender women has been published and adapted in several languages (12–15). However, there is still no validated Brazilian Portuguese version of a voice satisfaction questionnaire aimed at the population of transgender men.

In one of the pioneering studies to adapt the Transgender Woman Voice Questionnaire for the male gender, 145 transgender women and 83 transgender men participated and only the gender-specific words were modified. The questions were organized into three factors labeled as anxiety and avoidance, vocal identity, and vocal function. This may contribute to the development of shorter versions of the questionnaire, which can be useful for rapid screening of voice problems in transgender people undergoing HT (16).

The Transgender Woman Voice Questionnaire was adapted for the male gender, named Transsexual Voice Questionnaire for Female-to-Male (a-TVQ^FtM^), with validity and reliability analyses performed in a sample of 50 Turkish transgender men (27 naïve to HT and 23 undergoing HT) (17). The results showed strong internal consistency of the questionnaire, with a Cronbach’s alpha coefficient of 0.94 for anxiety and avoidance, 0.94 for vocal identity, and 0.92 for vocal function. The authors did not exclude any items and found a strong inverse correlation with overall voice-related quality of life scores (*r* = −0.863) and self-perception of male voice (*r* = −0.715) (*p* < 0.001). The a-TVQ^FtM^ scores were significantly reduced (i.e., increased voice satisfaction) in transgender men undergoing HT compared with those naïve to HT. The authors concluded that the instrument was adequate for use in the population of transgender men (17).

In Brazil, a team of speech therapists and researchers has developed a Trans Outpatient Vocal Care Protocol alongside a Voice Reassignment Program tailored for transgender individuals. The protocol’s first stage entails user referral; in the second stage, a comprehensive speech therapy evaluation is conducted, incorporating self-perception assessments and referrals for otorhinolaryngological examination. Based on the results, an individualized therapeutic approach is determined: either targeting the sound source or employing Protocol-Trans for vocal filtering. In the third stage, 12 vocal parameters are addressed monthly through both individual and group sessions with clients. The final stage involves follow-up referrals for both speech therapy and otorhinolaryngological re-evaluation. Notably, no specific vocal satisfaction or self-perception protocols designed explicitly for transmasculine voices were identified (18).

Indeed, as shown in a case study of a transgender man undergoing hormone therapy, characteristic female vocal traits were identified, including prolonged vowel sounds, rising intonation at the end of sentences, pitch variation, brief and infrequent pauses, high pitch, and a fundamental frequency (f0) within the female range. In this context, the authors recommend systematic voice assessments throughout testosterone treatment, as it may not consistently produce communication patterns perceived as male, and vocal instability is commonly observed (19).

As healthcare professionals caring for transgender and gender-diverse individuals in a public hospital setting, we recognized the need to develop protocols for accurately assessing the vocal satisfaction of trans men. Therefore, the purpose of our study was to adapt and apply the Transgender Man Voice Questionnaire in a sample of Brazilian transgender men and to investigate the relationship between voice satisfaction and duration of gender-affirming HT (GAHT).

## Materials and methods

The study was approved by the ethics committee of the institution (number 2018-0128). All participants were informed of the procedures and signed an informed consent form before inclusion in the study, in accordance with Resolution 466/12 of the Brazilian National Research Ethics Committee.

The study population was a convenience sample of transgender men participating in the Gender Identity Program (GIP), in a public hospital in Brazil, who agreed to the research objectives and provided written informed consent. Sociodemographic and clinical data were collected from the participants’ electronic medical records: age, smoking status, and type, completion, and duration of GAHT.

Inclusion criteria were transgender men aged 18–50 years with a diagnosis of gender dysphoria (according to DSM-V criteria) followed up within the GIP who were undergoing GAHT for at least 1 month but did not receive speech therapy or other treatment for voice deepening. Exclusion criteria were difficulty understanding the questionnaire, previous laryngeal surgery or hearing impairment that could affect communication, and incomplete medical records.

GAHT consisted of intramuscular injections of testosterone esters, with individualized intervals between applications to keep serum testosterone levels within the reference range for males and to achieve adequate clinical outcomes, including the development of sexual characteristics of the desired gender attribution and interruption of the menstrual cycle.

As there is no questionnaire to know the vocal satisfaction of Transgender Men, we adapted the Trans Woman Voice Questionnaire, translated and validated into Portuguese, with modifications related to gender (15). The questions were modified mainly at the heading (we added duration of GAHT) and the gender-specific words. We added a question: 31 (After GAHT, my voice became completely male), with the response options yes or no. In questions 32 and 33, asking participants to provide an overall rating of their voice (respectively, ‘Currently, my voice is:’ and ‘My ideal voice would sound:’), the order of the responses was reversed, starting with “very male” and ending with “very female.”

The answers are recorded on a 4-point Likert scale, as follows: 1 = never or rarely; 2 = sometimes; 3 = often; and 4 = usually or always (Supplementary Material). The total score ranges from 0 to 120, with higher scores indicating greater dissatisfaction with voice. Initially, we applied the questionnaire to five individuals in the group to verify the understanding of the questions, and possible suggested modifications. As no difficulties or suggestions were reported, we included these individuals in the final sample. The adapted questionnaire was answered individually (self-administered) during the final minutes of a GIP group session following a brief explanation about the purpose of the study. Any questions about the content of the questionnaire were resolved at the time of application. Participants had basic knowledge of voice production and voice care, as a speech therapist occasionally takes part in GIP group sessions.

The sample was categorized into smokers (current, occasional, or former) and non-smokers due to the impact of tobacco use on the voice.

Statistical tests were used to describe the sample, to decide the reliability of the questionnaire in analyzing satisfaction with voice in the study population, and to relate duration of GAHT with voice satisfaction. Split-half Spearman-Brown coefficient was analyzed by using the following strategies: first, the items were randomly divided into two equal halves and a scale mean was computed for each half, and then the two sets of scale means were correlated to estimate a split-half correlation, which was adjusted by the Spearman-Brown prophecy formula to calculate split-half reliability. This procedure was repeated 1,000 times, and the mean of the split-half correlations was taken as the best estimate of the reliability for a single item, whereas the mean of the split-half reliabilities was taken as the best estimate of the reliability for the composite of all items. The Multicon package version 1.6 of R was used.

The reliability of Cronbach’s alpha coefficient ranges from 0 to 1, and the internal consistency of scale items is often considered low for values below 0.70. In the present study, the reliability of Cronbach’s alpha coefficient was classified as follows: *α* ≤ 0.30, very low; 0.30 < *α* ≤ 0.60, low; 0.60 < *α* ≤ 0.75, moderate; 0.75 < *α* ≤ 0.90, high; and *α* > 0.90, very high.

## Results

The initial sample consisted of 33 self-identified transgender men. One man was excluded due to inconsistent data from the medical record and another because he had not started hormone treatment, bringing the total number of participants who completed the Transgender Man Voice Questionnaire to 31. Mean patient age was 30.13 ± 7.6 years, ranging from 19 to 49 years. The mean duration of GAHT was 29.7 ± 24.9 months, with testosterone cypionate being used in 95% and other long-acting testosterone and testosterone esters in the remaining participants.

Smoking was reported by 19.4% of the sample. All correlations performed showed no significant differences between smokers and non-smokers in terms of questionnaire total score or other study variables.

**Figure 1:**
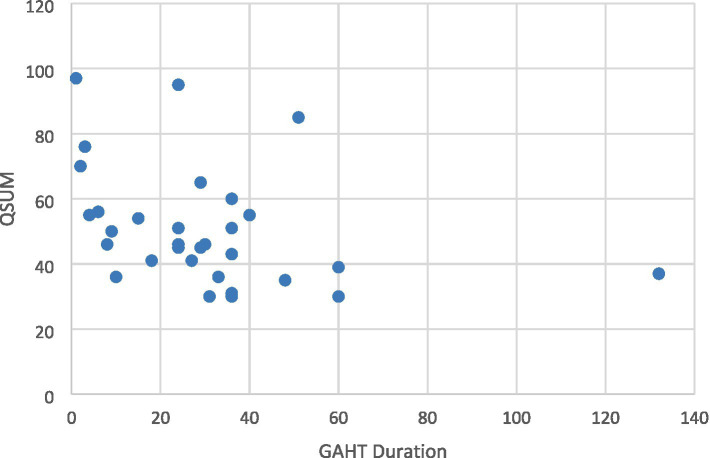
Correlation between questionnaire total score (QSUM) and gender-affirming hormone therapy (GAHT) duration.

The questionnaire mean total score was 51 ± 17.72, ranging from 30 to 97. In question 31, 58% participants reported that their voice sounded completely male after GAHT. In question 32, 48.4% answered that their voice is currently somewhat male, 29% that it is very male, 12.9% that it is neutral, and 9.7% that it is somewhat female. In question 33, 83.9% would like their voice to sound very male, 6.5% somewhat male, 6.5% neutral, and 3.2% somewhat female. There was a significant negative correlation between the questionnaire total score and duration of GAHT (*r* = −0.484, *p* = 0.006; Spearman’s test) (Table 1 and Figure 1).

**Table 1:** Correlation between questionnaire total score (QSUM) and gender-affirming hormone therapy (GAHT) duration.

			GAHT duration	QSUM
Spearman’s rho	GAHT duration	Correlation coefficient	1.000	−0.484^**^
		Sig. (2-tailed)		0.006
		*N*	31	31
	QSUM	Correlation coefficient	−0.484^**^	1.000
		Sig. (2-tailed)	0.006	
		*N*	31	31

**Correlation is significant at the 0.01 level (2-tailed).

We divided the Trans Man Voice Questionnaire into three factors, according to Bultynck et al. (16): anxiety and avoidance (11 questions: 2, 7, 8, 12, 13, 16, 17, 23, 25, 26, and 30), with a mean score of 17.42; vocal identity (8 questions: 3, 4, 6, 10, 19, 20, 24, and 28), with a mean score of 13.4; and vocal function (11 questions: 1, 5, 9, 11, 14, 15, 18, 21, 22, 27, and 29), with a mean score of 20.03 (Table 2).

**Table 2:** Questionnaire divided into three factors.

Descriptive statistics				
			Statistic	Std. Error
**Anxiety and avoidance**	Mean		17.42	1.244
	95% Confidence interval for mean	Lower bound	14.88	
		Upper bound	19.96	
	5% trimmed mean		16.84	
	Median		15.00	
	Std. Deviation		6.927	
	Minimum		11	
	Maximum		35	
**Vocal identity**	Mean		13.42	0.971
	95% Confidence interval for mean	Lower bound	11.44	
		Upper bound	15.40	
	5% trimmed mean		12.93	
	Median		12.00	
	Std. Deviation		5.408	
	Minimum		8	
	Maximum		29	
**Vocal function**	Mean		20.03	1.230
	95% Confidence interval for mean	Lower bound	17.52	
		Upper bound	22.54	
	5% trimmed mean		19.67	
	Median		20.00	
	Std. Deviation		6.848	
	Minimum		11	
	Maximum		37	

The questions with the highest score in item 4 (usually or always) were: 23 (77.4%), 17 (77.4%) and 10 (71%). Those with the lowest frequency in item 1 (never or rarely) were 8, 9, 10, 12, 13, 14, 17, 19, 23 and 27, all with 3.2%.

The questionnaire had a high level of internal consistency/reliability, with a Cronbach’s alpha coefficient of 0.95 for all items and a split-half Spearman-Brown coefficient of 0.96.

## Discussion

In the present study, we adapted and applied the Transgender Man Voice Questionnaire, previously validated in Portuguese for its female version (15). Our initial hypothesis that trans men with longer GAHT would have lower scores on the Trans Man Voice Questionnaire (i.e., greater voice satisfaction) and that the questionnaire would be a reliable tool for screening voice problems was accepted.

GAHT increases the muscle mass of the vocal folds with subsequent lowering of the f0. In general, throughout treatment, the larynx adapts to produce the voice sounds with a lower frequency, in the masculine range (4). In our sample, the longer the GAHT, the greater the satisfaction with voice as measured by the Trans Man Voice Questionnaire, and this result is consistent with previous findings (2–4, 6–8). However, this is the first study on voice satisfaction in a Brazilian Portuguese-speaking population of trans men.

Conversely, we found that many participants undergoing GAHT had voice complaints (questionnaire mean total score of 51), and only 31.58% reported that their voice sounded completely male. These data are consistent with those of a previous study reporting a rate of dissatisfaction with voice pitch of 79%, as dissatisfaction with gender-related voice characteristics, difficulties with controlling vocal gender presentation, and mismatch between desired gender assignment and gender assignments received from others (9). The fact that part of our sample (12.9%, 4 cases) was at the beginning of GAHT may have contributed to the increased dissatisfaction with voice, since it is known that the effects of GAHT on voice deepening are more robust after 6 months (6, 8). In addition, according to question 33, most participants (83.9%) would like their voice to sound very male.

The internal consistency/reliability scores of the Trans Man Voice Questionnaire showed that it can be applied to the population of trans men. Although not all study participants had voice complaints, a portion of this population does not obtain satisfactory results with HT alone and needs gender affirming voice therapy (1, 2). The questionnaire responses offer crucial insights to guide the creation of vocal and communication support programs for transgender individuals, particularly in identifying challenges such as difficulties with pitch range and variability (questions 11 and 29) and feelings of frustration when attempting voice modification (questions 15 and 16).

Vocal enhancement programs for trans individuals aims not only to modify specific aspects of voice quality but also to address all vocal attributes the person identifies as essential to their gender expression (18). In this context, the Trans Masculine Voice Questionnaire helps fill a gap in the literature and supports therapeutic listening, allowing speech therapists to gain a deeper understanding of the individual’s perception of their own voice.

The Trans Man Voice Questionnaire was organized into three factors, according to Bultynck et al. (16) producing similar minimum and maximum scores: anxiety and avoidance ranged from 11 to 35 vs. 11 to 44, vocal identity ranged from 8 to 29 vs. 8 to 32, and vocal function ranged from 11 to 37 vs. 11 to 44. Previous studies have also divided the instrument to facilitate medical planning, as a shorter version provides quick information and insights for voice therapy: the Voice Handicap Index is divided into functional, emotional, and physical aspects of voice disorders, while the TVQ^MtF^ is divided into two components (vocal functioning and social participation) (11, 20–22). We agree with Bultynck et al. (16) that a shorter version of the Trans Man Voice Questionnaire would facilitate its application as a screening tool in endocrinology offices and services. In addition, the authors believe that some questions may be less relevant for trans men, such as questions 11 and 16 related to pitch variation. In our study, however, these questions were scored high and, according to our clinical experience, complaints were observed mainly in voice professionals, such as difficulty in achieving high tones after GAHT. Further research is needed to investigate whether complaints of pitch variation in trans men are relevant.

Coleman et al. (2) reports that studies on tone reduction surgeries are rare. However, two studies describe a statistically significant reduction in fundamental frequency post-surgery, due to continued dissatisfaction with hormonal treatment (16, 25). In clinical research, it is important to address potential variables not covered in the questionnaire.

In addition, based on the most frequently asked questions answered by transgender men, our clinical practice and questions with redundant topics (29 and 11), we suggest removing questions 8, 10, 12, 13, 14, 17, 19, 23, 27, and 29 for the elaboration of a reduced questionnaire for screenings, in a future study. Another suggestion would be to modify question 1, with the aim of identifying difficulties with projection and decreased vocal intensity, common complaints observed in transgender men. According to Hardy et al. (23) there are possible benefits in modifying the sound pressure level when a masculine identity is desired.

Gender dysphoria is currently understood as a result of sociocultural processes such as stigmatization, pathologization, coping, and resilience (24). Speech therapists who provide services to transgender individuals must also take these factors into consideration when administering the questionnaire.

## Limitations

The study has some limitations such as small sample size and the characteristics of the study population, consisting of transgender men treated within a gender identity program in a public hospital in Brazil who often seek the service for gender-affirming surgery. The study used a cross-sectional design, assessing participants’ voice satisfaction and hormone therapy duration at a single time point, which does not reveal causality or long-term effects. Additionally, the lack of a comparison group specifically transgender men who have not undergone hormone therapy—limits the study’s findings.

Another aspect is that relying on self-reported vocal satisfaction may introduce social desirability bias and cognitive distortions, which can impact data accuracy. However, this study includes new data on the voice assessment of Brazilian transgender men, an under-represented population in studies investigating transgender health care.

According to Coleman et al. (2), priority should be given to understanding that gender does not exist in isolation, but intersects with other aspects of human diversity such as: First Nation status, ethnicity/race, sexuality, disability/ability, faith/religion/spirituality. Thus, the human voice is complex and other factors not investigated in this questionnaire may influence vocal satisfaction results.

## Conclusion

The Transgender Man Voice Questionnaire demonstrates reliable psychometric properties, making it suitable for use among Portuguese-speaking transgender men. Additionally, the instrument effectively indicates that extended duration of GAHT is associated with a more masculine-sounding voice and increased voice satisfaction. This tool may serve as a valuable screening instrument in clinical practice and as a complementary resource for planning vocal enhancement programs. However, it is recommended that the questionnaire be used and interpreted with caution, supplemented by data obtained from anamnesis and clinical evaluation. Future research with larger samples of transgender men could further validate this tool, and therapeutic listening might support the refinement of the protocol.

## Data Availability

The original contributions presented in the study are included in the article/supplementary material, further inquiries can be directed to the corresponding author.
